# Efficacy of botulinum toxin type A in myogenic temporomandibulardisorders: a single arm, prospective, pilot study

**DOI:** 10.3389/fresc.2025.1737706

**Published:** 2025-12-19

**Authors:** Bruno Macedo de Sousa, Nansi López-Valverde, Carolina Ferreira, André Mariz de Almeida, Antonio López-Valverde, José A. Blanco Rueda

**Affiliations:** 1 Institute for Occlusion and Orofacial Pain Faculty of Medicine, University of Coimbra, Polo I-Edifício Central Rua Larga, Coimbra, Portugal; 2 Department of Surgery, University of Salamanca, Salamanca, Spain; 3 Instituto de Investigación Biomédica de Salamanca (IBSAL), Salamanca, Spain; 4 Egas Moniz Center for Interdisciplinary Research, Egas Moniz School of Health & Science, Almada, Portugal

**Keywords:** temporomandibular joint disorders, myofascial pain, botulinum toxin type A, pain management, quality of life

## Abstract

**Background:**

Myogenous temporomandibular disorders (TMDs) is commonly associated with myofascial pain and functional limitations. Botulinum toxin type A (BoNT-A) has shown potential in relieving chronic muscular pain.

**Methods:**

In this single arm, prospective, pilot study, 25 patients diagnosed with myofascial TMDs received a single bilateral intramuscular injection of 50 units of BoNT-A. Assessments using the Visual Analog Scale (VAS), Chronic Pain Index (CPI), Jaw Functional Limitation Scale (JFLS-8), and Oral Health Impact Profile (OHIP-14) were performed at baseline, 6 weeks, and 12 weeks post-treatment.

**Results:**

Significant reductions in pain (VAS: 6.84 ± 1.03 to 0.84 ± 0.94) and CPI (61.87 ± 8.12 to 9.86 ± 4.46) were observed. Functional improvement (JFLS-8: 5.82 ± 0.56 to 1.08 ± 0.51) and enhanced quality of life (OHIP-14: 24.56 ± 8.39 to 5.96 ± 2.79) were also significant. No adverse effects were reported.

**Conclusions:**

BoNT-A showed promising results in reducing pain and improving function in patients with myofascial pain in Temporomandibular Disorders (M-TMDs). However, the single-arm design, small sample size, and short follow-up limit causal inference and external validity. These findings should be interpreted as preliminary.

**Clinical Trial Registration**: Clinicaltrials.gov, identifier NCT05651256.

## 1 Introduction

Temporomandibular disorders (TMDs) are a widely studied topic encompassing a variety of musculoskeletal conditions affecting the temporomandibular joint (TMJ), masticatory muscles, and associated structures. Among the most common conditions are myofascial pain, joint disc displacement, and degenerative joint diseases. Diagnosis is often established using standardized systems, such as the Diagnostic Criteria for TMDs (DC/TMDs), which are universally regarded as the gold standard in scientific research (1). These criteria are organized into two main axes: Axis I, which includes diagnoses based on specific clinical criteria for the most common painful conditions and intra-articular disorders, and Axis II, which assesses biopsychosocial dimensions such as anxiety, depression, stress, and pain-related disability, allowing for a broader and more integrated analysis of the impact of the disorder on the patient's life (2). TMDs present with a wide range of signs and symptoms, including pain in the masticatory muscles, which may radiate to the neck and head; headaches; hearing disorders; tooth wear; limited jaw mobility; muscle hypertrophy; and inflammatory processes or joint noises during jaw movements (3). They are considered the leading cause of non-dental/periodontal orofacial pain and affect people of all ages, although their prevalence is highest in young adults between the ages of 20 and 40. There is a marked female predominance, with a ratio of approximately four women for every man affected. Studies indicate that approximately 31% of adults experience TMDs at some point in their lives. Beyond its high prevalence, the condition can significantly affect quality of life, interfering with essential activities such as chewing, speaking, and swallowing, as well as simple gestures such as yawning (4–6).

The etiology of TMDs is multifactorial, reflecting the interaction between biological, psychological, and social factors. Biologically, it may be associated with genetic predisposition, hormonal changes, pain modulation, and overall physical health (7). Psycho-logically, individual beliefs, anxiety, fear, depression, sleep disorders, and mood changes play a significant role (8). Socially, interpersonal relationships, culture, socioeconomic status, and occupational conditions may influence the onset and progression of TMDs. The interaction between these biopsychosocial factors not only contributes to its manifestation but also affects treatment response, particularly in cases with associated psychological comorbidities such as anxiety and depression. TMDs imposes both individual and societal burdens. Functional limitations and chronic pain substantially reduce patients' quality of life. From an occupational perspective, the condition is associated with increased absenteeism, higher healthcare expenditures, and persistent consultation of multiple healthcare providers (9).

The treatment of TMDs requires a multidisciplinary approach, initially focused on conservative and reversible options aimed at alleviating symptoms without resorting to invasive or irreversible interventions. Treatment usually begins with patient education and learning self-care strategies. These include controlling oral parafunctions (e.g., avoiding teeth clenching or excessive chewing gum use), adopting good sleep hygiene, performing therapeutic exercises, and relaxation techniques. The use of intraoral appliances, such as stabilization splints, is another common strategy for short-term pain relief, although their effectiveness, compared to placebo, remains a subject of debate (10).

Physical therapy also plays a key role, incorporating mobilization and strengthening exercises that have been shown to be effective in reducing pain and improving joint function (11). In more complex cases, pharmacotherapy may be used, employing muscle relaxants, anti-inflammatories, or other centrally and peripherally acting agents (12, 13).

Psychological interventions, such as cognitive behavioral therapy (CBT), have demonstrated long-term benefits, especially in patients with significant psychological symptoms. These approaches, which may include biofeedback, relaxation techniques, and counseling, complement physical treatments by addressing the emotional and social factors that contribute to dysfunction (14). Other strategies, such as acupuncture and dry needling, have proven effective in relieving muscle pain. Intra-articular injections are more suitable for joint pain. Invasive procedures, such as arthrocentesis and surgery, are reserved for severe or treatment-resistant cases (15, 16). For muscle conditions that do not respond to conventional treatments, new approaches have been investigated, such as the use of botulinum toxin type A (BoNT-A). It is a powerful neurotoxin produced by the gram-negative spore-forming bacterium Clostridium botulinum and has become a common treatment for TMDs in recent years. This therapy has shown potential for relieving chronic myofascial pain and other associated muscle conditions, offering hope to patients with persistent and refractory symptoms (17, 18). However, there is no consensus on the effects of BoNT-A in the treatment of painful TMDs, nor on the effects of BoNT-A application in the masticatory muscles of people with bruxism, despite its increasing use in dentistry (19–22). For a time, it was thought that the analgesic effect of botulinum toxin type A (BoNT-A) was due to its muscle-relaxing effect (23). However, recent studies in neuropathic pain models have demonstrated that it has an analgesic effect independent of muscle relaxation through a dissociative effect between the duration of muscle relaxation and the duration of pain relief (24). However, despite having proven effective in treating various conditions, including TMDs, concerns remain about possible adverse effects, particularly on mandibular bone health (25, 26). Studies show that BoNT-A injections into the masticatory muscles can reduce muscle fiber size and alter fiber type proportions, increasing the proportion of type IIb fibers, which have lower fatigue resistance. The findings indicate that a single BoNT-A injection temporarily reduces masticatory function and causes degenerative changes in muscle tissue and bone structure, including alveolar bone resorption, lasting at least four weeks (25, 27). However, there is also no consensus regarding bone-related aspects: repeated injections of BoNT-A appear to exacerbate bone loss compared to single applications; nevertheless, there are no conclusive reports from high-quality studies on this aspect, with most studies being of suboptimal methodological quality and indicating small-magnitude bone changes, which should be interpreted with caution (28). Therefore, although BoNT-A remains a valuable therapeutic tool, it is essential to carefully evaluate its potential risks and benefits in the clinical treatment of TMDs.

The objective of this pilot study was to evaluate the effects of BoNT-A on M-TMDs, as well as to assess its impact on mandibular function and quality of life in treated individuals. This study also analyzed adverse effects to evaluate the safety of BoNT-A in patients with TMDs.

## 2 Methods

This is a prospective, multicenter, single-group pilot study conducted in collaboration with two academic institutions: The Faculty of Medicine of the University of Coimbra (Portugal) and the Egas Moniz University Institute (Portugal). Its implementation was authorized by the Ethics Committee of the Faculty of Medicine of the University of Coimbra, which issued a favorable opinion at its meeting on September 14, 2023, under file number CE-090/2023. The study is also registered on Clinicaltrials.gov under identifier NCT05651256. All participants included in the study signed informed consent, in accordance with the ethical requirements stipulated by the aforementioned committee.

The study was conducted and reported in accordance with the Strengthening the Reporting of Observational Studies in Epidemiology (STROBE) guidelines, which are suitable for single-arm interventional designs without a control group.

### 2.1 Participants

No formal sample size calculation was performed due to the exploratory and pilot nature of this study. The number of participants was determined based on the estimated recruitment feasibility within the study period and the sample sizes used in similar published studies on botulinum toxin for myogenic temporomandibular disorders. This approach is consistent with previously reported exploratory single-arm trials.

**Figure 1:**
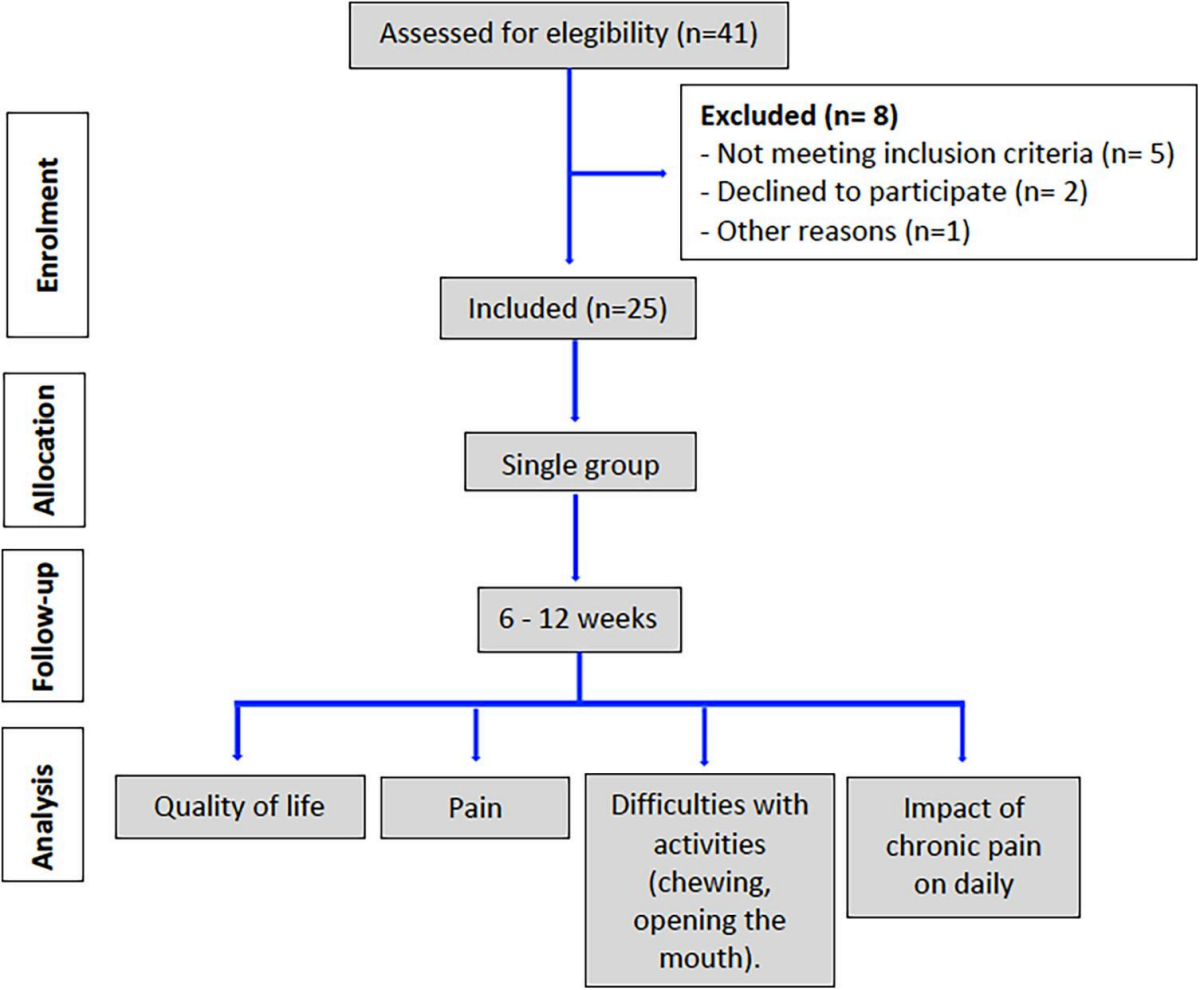
Flow chart of progress through the phases of the study according to the CONSORT statement 2010 (29).

The sample for this study consisted of 25 participants, of whom 21 were women and 4 were men, aged between 23 and 69 years. The mean age was 53.60 ± 12.25 years. The total study period lasted 12 weeks and took place between November 2024 and February 2025. Participant selection and study phases are presented in a flow chart (Figure 1).

### 2.2 Patient description; inclusion and exclusion criteria

The patients recruited came from the clinics of the two participating institutions. The study exclusively included participants with a confirmed diagnosis of myofascial pain, according to DC/TMD, lasting more than three months and whose pain had not responded to previous conservative treatment.

Participants underwent an initial screening visit 1–2 weeks prior to the baseline assessment. During the screening visit, eligibility was confirmed, medical history was obtained, and DC/TMD diagnostic procedures were completed. All participants underwent standardized clinical assessment including structured history-taking, muscle palpation of the masticatory muscles, and pain localization tests. Diagnostic criteria for myalgia and myofascial pain were applied following DC/TMD definitions.

Exclusion criteria were previous surgery or arthrocentesis of the temporomandibular joint; surgery in the cervicofacial region in the previous six months; active follow-up in a Pain Unit at the time of inclusion; history of previous successful treatment; history of previous treatment with BoNT-A.

All clinical examinations were performed by a single clinician trained in the DC/TMD protocol. Calibration was achieved through joint assessment of pilot cases prior to study initiation, following the recommendations of Schiffman et al. (2014) (1). To minimize confounding, the use of new analgesic medications, muscle relaxants, physiotherapy, occlusal splints, or any other TMD-related interventions was not permitted during the follow-up period.

Axis II (psychosocial) measures were not collected, as the study focused on somatic (Axis I) diagnostic outcomes. This omission is acknowledged as a limitation regarding the assessment of psychosocial comorbidities.

### 2.3 Treatments

**Figure 2:**
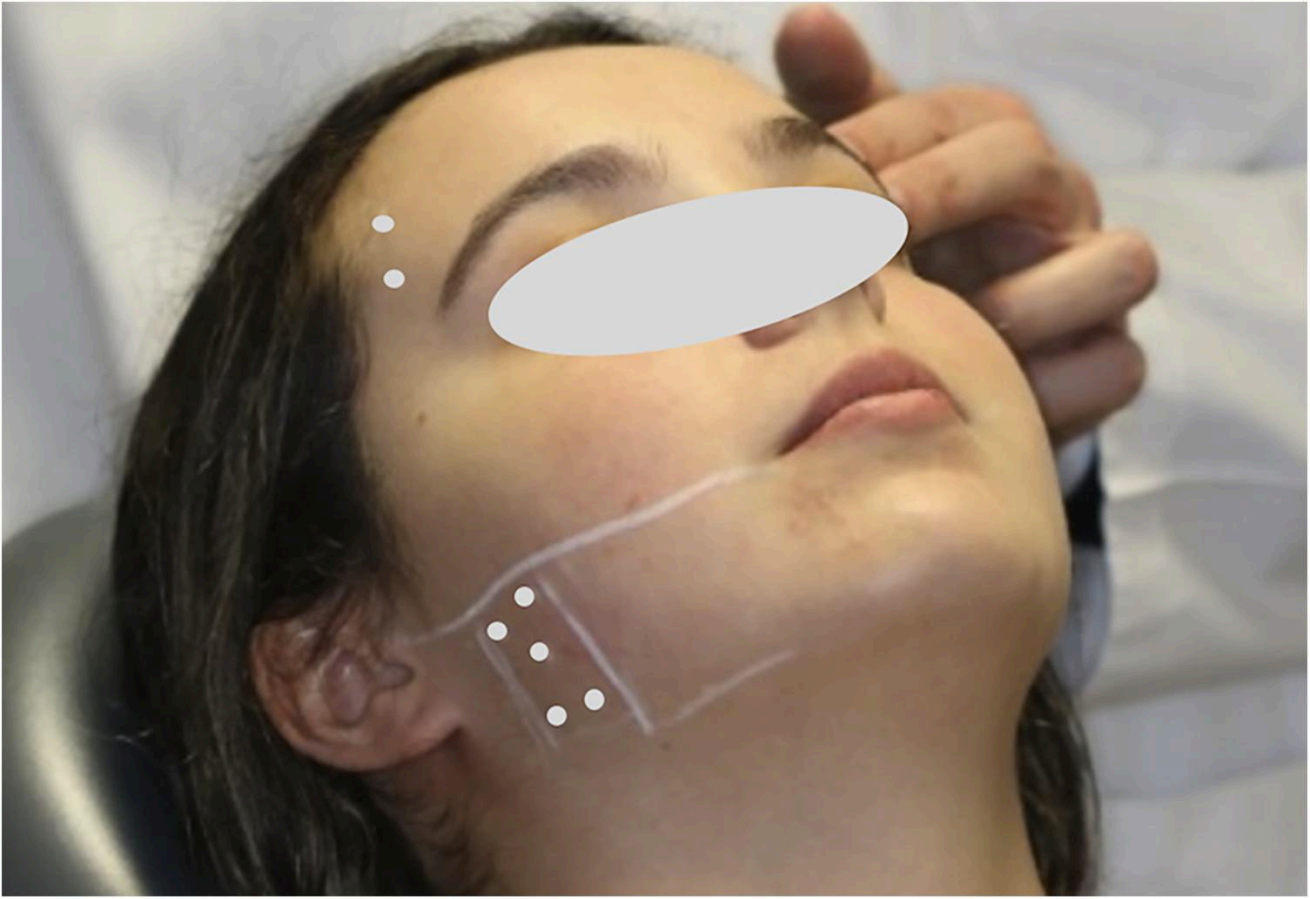
Marking of the injection points with the aid of muscle palpation and contraction induced by dental occlusion.

**Figure 3:**
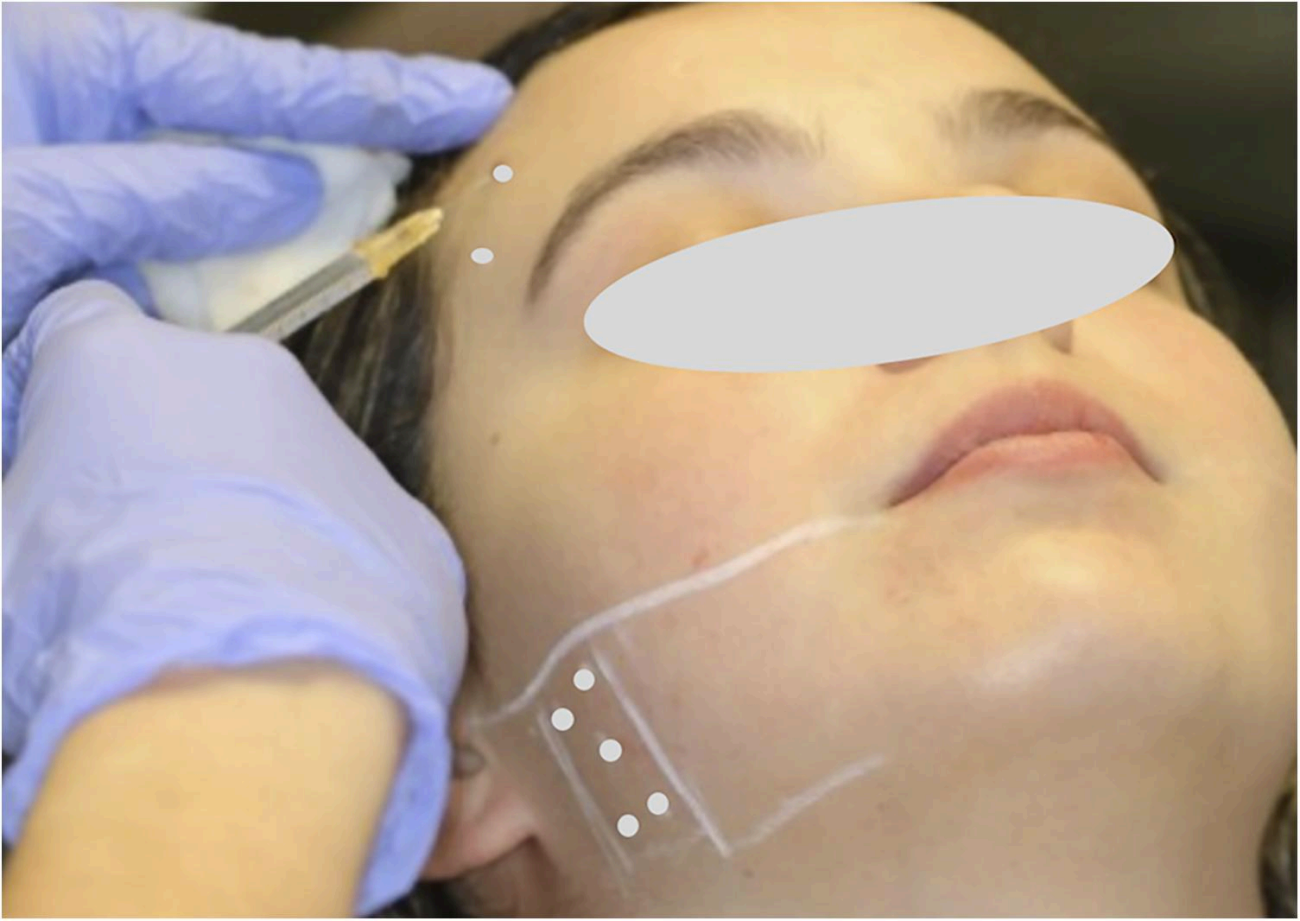
Administration of 5 units of BoNT-A at each temporal point and 3 units at each masseter point.

The therapeutic protocol consisted of a single session of bilateral intramuscular injections of BoNT-A, with a total of 50 units (Botox®, Allergan, Irvine, California, USA), previously reconstituted with 0.9% isotonic saline solution. The injection sites were identified based on muscle palpation and contraction induced by dental occlusion, according to a standardized anatomical pattern. Injections were performed using a 1 mL Luer-lock syringe with a 30-gauge, 13 mm needle (0.3 mm × 13 mm). In the anterior temporal muscle, two injection sites were defined in its anterior portion, with 5 units administered per site. In the masseter muscle, five injection points were arranged around a central point, with 3 units administered per point (Figures 2,3). Injections were performed bilaterally, with a total of 25 units per side of the face (30).

The lateral pterygoid was deliberately excluded from the injection protocol due to its deep location, proximity to major vascular structures, and higher risk of toxin diffusion to adjacent tissues such as the pterygoid venous plexus, maxillary artery, or extraocular muscles. Injection of this muscle generally requires ultrasound guidance to ensure safe placement, which was not part of the standardized protocol used in this study. To prioritize safety and reproducibility across clinical settings, injections were therefore restricted to the masseter and anterior temporalis muscles, which are easily accessible and commonly targeted in M-TMD research.

No ultrasound guidance was used; instead, muscle palpation and visual anatomical cues were employed to ensure accuracy. All procedures were performed under aseptic conditions, and patients were advised to avoid massage or strenuous activity involving the injected muscles for 24 h post-procedure.

### 2.4 Adverse events

Adverse events (AEs) were defined according to the International Council for Harmonisation Good Clinical Practice (ICH-GCP) guidelines as any untoward medical occurrence following BoNT-A administration, irrespective of causal relationship to treatment.

AEs were actively solicited at each follow-up visit (weeks 2, 6, and 12) and through scheduled telephone interviews conducted one week after injection. Patients were specifically questioned regarding local reactions (e.g., pain, edema, bruising, muscle weakness, asymmetry) and systemic symptoms.

An AE was defined as any untoward medical occurrence following BoNT-A injection, irrespective of its causal relationship to treatment. Serious adverse events (SAEs) were defined as any event resulting in hospitalization, persistent disability, or life-threatening condition.

All AEs were recorded in standardized case report forms, graded according to severity (mild, moderate, severe), and assessed for possible relation to BoNT-A administration.

### 2.5 Measuring tools

To conduct a comprehensive assessment of TMDs and response to treatment, a set of validated instruments was used to analyze different dimensions of the patient experience. Pain was assessed using the Visual Analog Scale (VAS), a simple and widely recognized tool that allows patients to indicate pain intensity on a scale from 0 (no pain) to 10 (maximum pain) (31). The VAS is particularly useful for tracking pain over time and response to treatment. Complementarily, the 14-item Oral Health Impact Profile (OHIP-14) was used to measure the impact of oral health on quality of life, covering physical pain, functional limitations, psychological distress, and social impact (32, 33). To quantify specific functional limitations of the jaw, the 8-item Jaw Functional Limitation Scale (JFLS-8) was applied, which assesses difficulties in activities such as chewing, opening the mouth, and speaking, thus reflecting the degree of functional impairment experienced in daily life (34). In addition, the Chronic Pain Inventory (CPI) was used to characterize chronic pain, assessing not only the intensity and frequency of pain, but also its impact on daily activities, interpersonal relationships, and productivity, parameters that are particularly relevant to myofascial pain (35).

To support clinical interpretation of the outcomes, previously validated minimal clinically important differences (MCID) thresholds were applied. For pain intensity on the VAS, an MCID of 1–2 points is widely accepted for chronic musculoskeletal pain. For the OHIP-14, a decrease of 5–6 points represents a clinically meaningful improvement in oral health-related quality of life. For the JFLS-8, a change of approximately 4–8 points has been reported as clinically relevant for functional limitation in the jaw (1, 34).

These thresholds were used to interpret the magnitude of improvement observed in this study.

The combination of these instruments allows for a multidimensional analysis of TMDs, covering pain, jaw function, and quality of life, which are key aspects for a rigorous evaluation of treatment efficacy.

All outcome measures were collected by the same examiner who conducted the baseline evaluations. Due to the single-arm design and longitudinal nature of the study, blinding of the examiner to the treatment timepoint was not feasible. Nevertheless, standardized assessment procedures were applied consistently across all timepoints to minimize measurement variability.

### 2.6 Statistics

The collected data were organized in Microsoft Excel® spreadsheets and subsequently analyzed using GraphPad Prism® software version 9.5.1 (GraphPad Software, San Diego, CA, USA). Sample normality was verified using the Shapiro–Wilk test.

Statistical analyses were performed using IBM SPSS Statistics version v26 platform (IBM Corp., Armonk, NY, USA). Normality of data distribution was assessed with the Shapiro–Wilk test. For normally distributed variables, changes across timepoints were analyzed using repeated-measures ANOVA, followed by Bonferroni-adjusted pairwise comparisons. For non-normally distributed data, the Friedman test was applied, with Wilcoxon signed-rank tests for *post-hoc* comparisons.

Effect sizes were reported as partial eta-squared (*η*^2^) for ANOVA and r for nonparametric tests, with corresponding 95% confidence intervals (CIs) where applicable.

Missing data were handled by listwise deletion, as fewer than 5% of data points were incomplete. Statistical significance was set at *p* < 0.05 (two-tailed) after correction for multiple comparisons.

## 3 Results

### 3.1 Results for pain

All analyses were conducted according to the predefined statistical plan. Effect sizes and 95% confidence intervals are reported to facilitate interpretation of clinical relevance.

**Figure 4:**
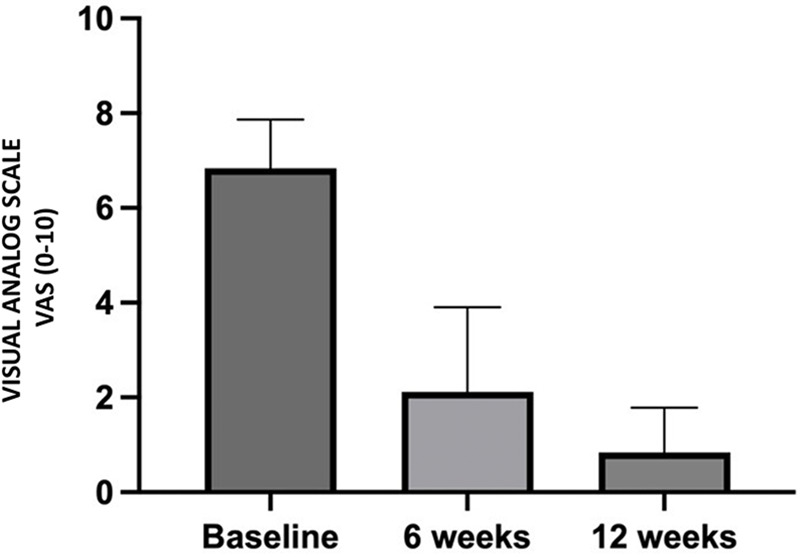
Perceived pain intensity reported by patients during the study period.

Statistical analysis revealed a significant reduction in pain intensity after BoNT-A administration. On average, the Visual Analog Scale (VAS) score decreased from 6.84 ± 1.03 at the start of the study to 2.12 ± 1.79 six weeks after treatment (*p* = 0.0002). Twelve weeks after treatment, the VAS score decreased to 0.84 ± 0.94 (*p* < 0.0001). Comparison of the mean pain scores at weeks 6 and 12 also revealed a statistically significant difference (*p* = 0.0400) (Figure 4).

### 3.2 Results for quality of life

**Figure 5:**
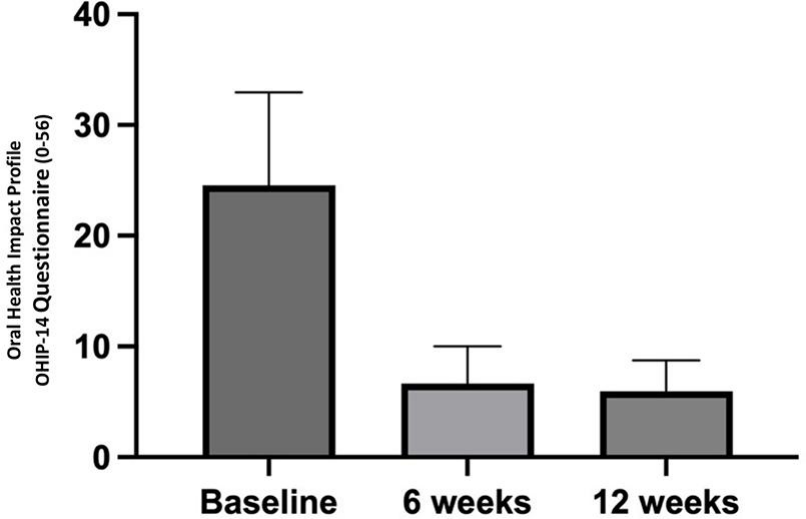
Patients’ perceived quality of life throughout the study period.

In terms of quality of life, assessed using the Oral Health Impact Profile (OHIP-14), the mean score decreased from 24.56 ± 8.39 at the start of the study to 6.64 ± 3.37 at six weeks (*p* < 0.0001) and to 5.96 ± 2.79 at twelve weeks (*p* < 0.0001). The difference between the values at weeks 6 and 12 was not statistically significant (*p* > 0.9999) (Figure 5).

### 3.3 Results for mandibular functional limitation

**Figure 6:**
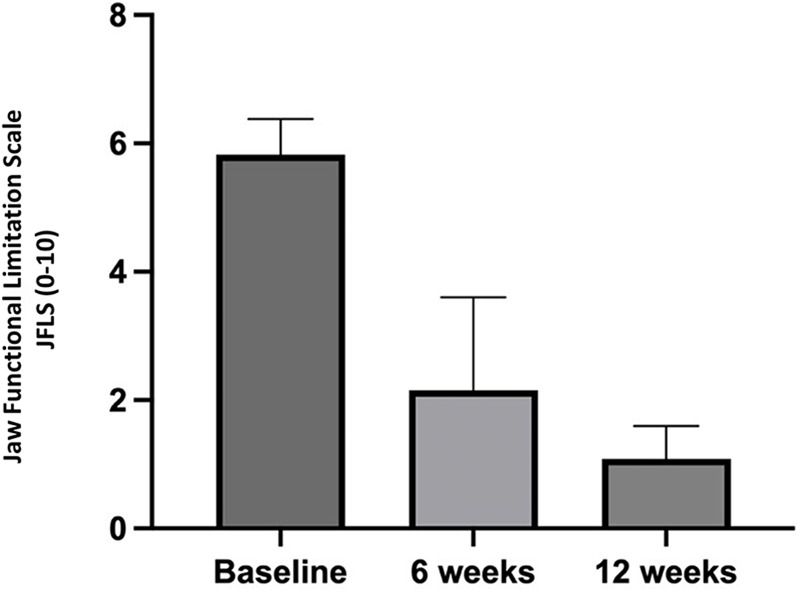
Patients’ perceived mandibular functional limitation throughout the study period.

Regarding mandibular functional limitation, measured using the Jaw Functional Limitation Scale (JFLS-8), a progressive improvement was observed, with a decrease in the mean score from 5.82 ± 0.56 before the intervention to 2.15 ± 1.45 after six weeks (*p* = 0.0001) and to 1.08 ± 0.51 after twelve weeks (*p* < 0.0001). The comparison between the values at weeks 6 and 12 showed no statistical significance (*p* = 0.1431) (Figure 6).

### 3.4 Results for chronic pain

**Figure 7:**
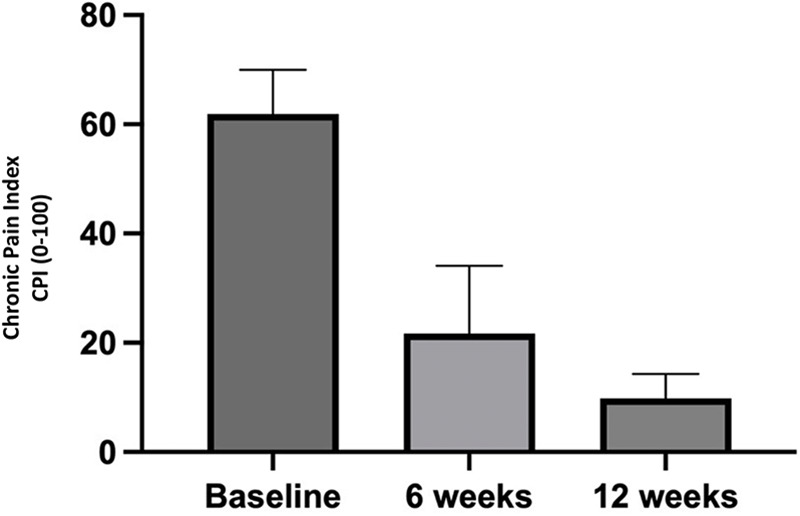
Perception of chronic pain by patients throughout the study.

As for the impact of chronic pain measured by the Chronic Pain Inventory (CPI), the mean score decreased from 61.87 ± 8.12 at baseline to 21.73 ± 12.37 at six weeks (*p* = 0.0016), and 9.86 ± 4.46 at twelve weeks (*p* < 0.0001). The comparison between the two post-intervention time points also showed a significant difference (*p* = 0.0016) (Figure 7).

No statistically significant correlations were observed between pain intensity (VAS) and quality of life (OHIP-14) at any of the assessment points: baseline (r = 0.1412; *p* = 0.5007), six weeks (r = −0.2073; *p* = 0.3200), and twelve weeks (r = 0.0291; *p* = 0.8901).

### 3.5 Adverse events communication

None of the patients reported AE during or after treatment.

## 4 Discussion

In the present pilot study, a clinically significant reduction in pain was observed in individuals with M-TMDs, both on the VAS and CPI, at 6 and 12 weeks after BoNT-A administration. These findings indicate a favorable short-term response to BoNT-A, supporting its potential role in pain control in cases of M-TMDs, while acknowledging that the single-arm design precludes causal inference. The absence of significant adverse events further reinforces the apparent safety profile of the intervention within the short follow-up period.

The marked decrease in VAS scores, already evident at six weeks, indicates an early analgesic effect of BoNT-A. These results are consistent with those published by Le Victor et al. and Jadhao et al. (19, 36), who, in two randomized studies, reported substantial reductions in myofascial pain after a single administration of the toxin, with effects that persisted, in some cases, for up to six months. Guarda-Nardini et al. (37) in a pilot study in patients with bruxism, supported treatment with BoNT-A to improve pain at rest and during chewing. Existing scientific evidence contextualizes and reinforces our findings. A double-blind randomized clinical trial conducted by De la Torre Canales et al. (38) demonstrated that BoNT-A promotes significant pain reduction and perceived improvement in patients with refractory M-TMDs, with greater efficacy than placebo. Although the participants in our study did not meet the criteria for refractoriness, com-parable results were observed, suggesting that BoNT-A may also be effective in the early stages of the disease, thus broadening its therapeutic potential. Montes-Carmona et al. likewise reported significant improvements in cases of masticatory myofascial pain, both localized and referred (39). The similarity of their findings with those of the present study is particularly relevant, given that our participants presented with both pain patterns, reinforcing the applicability of BoNT-A in different manifestations of M-TMDs. Gonzalez-Perez et al. (40) also highlighted a good therapeutic response with the use of BoNT-A in the treatment of chronic masticatory myalgia, with high efficacy and safety in long-term pain reduction.

However, contemporary systematic reviews and meta-analyses have emphasized substantial heterogeneity across RCTs regarding injection protocols, total dose, and target muscles. Most controlled studies have used per-muscle doses between approximately 10 and 75 U, most often distributed in three to six injection points per muscle, targeting the masseter and/or temporalis. Our dosing regimen (50 U total; 25 U per side, five points in the masseter and two in the anterior temporalis) therefore lies within the lower–moderate range of the doses commonly used in recent RCTs and systematic reviews (36, 37, 39, 41). This moderate-dose, multi-point approach was deliberately selected to balance potential analgesic benefit with safety, particularly in light of recent concerns about dose-related adverse effects.

The early pain improvement may partly account for the functional gains observed in JFLS-8 assessments, supporting the theory that pain reduction precedes and facilitates mandibular movement recovery (42). This tool, used in our study and designed specifically to assess jaw function, revealed significant improvements at 6 and 12 weeks, reflecting progressive functional recovery, in line with the results of De la Torre Canales et al. (43). However, despite the improvements observed in pain and jaw function, the effects of BoNT-A on maximum mouth opening remain controversial. Studies such as those by Guarda-Nardini et al., De Carli et al. and Kim et al. did not observe significant differences between the treated groups and the control groups (37, 44, 45). In contrast, Nixdorf et al. reported a significant reduction in mouth opening, albeit with a high risk of bias due to dropout rates (46). However, a recent meta-analysis by Li et al. demonstrated a significant increase in pain-free opening after one month and unassisted opening at 1 and 6 months (47). Such discrepancies between studies may be related to methodological differences, the initial characteristics of the participants, or the use of complementary interventions. The results obtained on the OHIP-14 scale after treatment with BoNT-A indicate a significant improvement in oral health-related quality of life, corroborating the psychosocial benefits of this intervention in people with myofascial pain. These findings are consistent with those of De la Torre Canales et al., who demonstrated that BoNT-A not only reduces pain but also contributes to the reduction of psychological comorbidities frequently associated with TMJ (48). Therefore, the scores obtained in the OHIP-14 can be understood as a direct consequence of symptom relief, as several studies suggest that reduced pain intensity is closely related to a lower functional and psychosocial impact in patients with TMDs (49, 50). Another relevant aspect is that the observed benefits may also be associated with indirect effects of BoNT-A on mental health. According to the literature, this toxin appears to have a positive influence on variables such as anxiety and depression (51, 52), factors which, when present, amplify the perception of disability and worsen the discomfort associated with orofacial limitations (53). The hypothesis of a central action of BoNT-A, specifically in the modulation of limbic pathways involved in emotional processing (54, 55), is a plausible explanation for the overall improvement described by patients, even in areas assessed by the OHIP-14 tool, such as psychological distress and functional limitation (56).

Regarding BoNT-A application protocols for the treatment of M-TMDs, several recent systematic reviews (including those by Li et al., De la Torre Canales et al., Delcanho et al. and Kharraz et al. (47, 48, 57, 58) have consistently demonstrated a lack of consensus. These studies highlight the heterogeneity in the doses used (50–300 units), the muscles selected for injection, and the administration techniques adopted. Given this methodological diversity, our study opted for a therapeutic protocol that prioritizes safety without com-promising efficacy, based on the most recent scientific evidence. The choice of a moderate dose of 50 units (25 U per hemisphere) was based on several factors. First, the review by De la Torre Canales et al. (48) warns of the possible adverse effects associated with higher doses, such as muscle atrophy, bone alterations, and significant reduction in masticatory strength. These data are corroborated by De la Torre Canales et al., who demonstrated in a sample of 100 women that higher doses are not associated with greater efficacy, but with a higher incidence of adverse effects. In addition, they suggested that a single administration of reduced doses can significantly improve pain without causing adverse effects (59). The administration technique used with multiple injection sites (five in the masseter and two in the anterior temporalis), with reduced doses per site, was designed to optimize toxin distribution. This approach promotes greater dispersion of the active substance while maintaining the same total dose, ensuring more homogeneous distribution within the target muscle. By avoiding high local concentrations, this technique also reduces the risk of local adverse effects and contributes to the observed safety profile (60). The selection of the masseter and anterior temporalis muscles as therapeutic targets is based on anatomical and accessibility criteria. These muscles are easily located by palpation, unlike the pterygoid muscle, which requires ultrasound guidance for safe application (61). This choice increases the reproducibility of the protocol in clinical settings and minimizes the risk of accidental injections into adjacent structures. The clinical relevance of this muscle selection is likewise supported by the review by De la Torre Canales et al., which highlights the central role of these muscles in the pathophysiology of M-TMDs (62). The timing of the assessments (6 and 12 weeks after treatment) was strategically chosen to capture both the peak action and duration of the therapeutic effects. This decision was based on the literature describing the peak muscle action of BoNT-A between 1 and 4 weeks, with effects that can persist for 3–4 months (63, 64). By including these two assessments, the present study offers a comprehensive perspective on the temporal profile of the therapeutic response.

Recent systematic reviews and experimental studies have also raised important considerations about the structural safety of BoNT-A for masticatory muscles. Although clinical studies generally report only mild and transient adverse events, several imaging and histological investigations have demonstrated masseter muscle atrophy and mandibular bone cortical thinning following repeated or high-dose injections. Animal models and human imaging studies suggest that BoNT-A–induced muscle inactivity may reduce mechanical loading on the mandible, leading to localized bone remodeling and density loss. These potential effects appear to be dose- and time-dependent and may partially reverse after several months, but long-term data in humans remain scarce. Consequently, the apparent short-term safety observed in this and similar studies should be interpreted with caution, and further randomized, placebo-controlled trials with longitudinal imaging follow-up are warranted to assess bone and muscle outcomes more comprehensively (7, 14, 65, 66).

Despite the promising results of this multicenter pilot study, some limitations must be acknowledged. The relatively small sample size and short follow-up further restrict the generalizability of the findings. The administration of BoNT-A in a single session prevented the evaluation of cumulative or prolonged effects of treatment, which would be relevant for defining more sustainable therapeutic protocols. The absence of a placebo control group is another limitation, as it makes it difficult to distinguish between the specific effects of the intervention and possible placebo effects. In addition, the sample consisted exclusively of participants recruited from academic institutions, which may limit the generalizability of the results to a broader population with M-TMDs.

Furthermore, no direct comparisons were made with other currently used therapeutic approaches, preventing conclusions about the relative efficacy of BoNT-A. It is also important to note that, despite the use of validated tools, the data collected were based on the subjective perceptions of participants, which may introduce bias in the interpretation of results.

In light of these methodological constraints, the present findings should be considered preliminary evidence supporting the potential benefit of BoNT-A in myogenous TMDs. Confirmation in larger randomized, placebo-controlled studies with longer follow-up and standardized imaging and functional endpoints is essential. Future research should also systematically monitor muscle thickness and mandibular bone density to clarify the structural safety profile of this therapy.

These limitations underscore the need for future studies with larger samples, adequate control groups, and long-term follow-up. It is also essential to develop standardized treatment protocols and clear clinical guidelines for the application of BoNT-A in M-TMDs. In addition, studies evaluating the economic feasibility of this intervention (cost/benefit ratio) should be conducted to determine the cost-effectiveness of its clinical use.

## 5 Conclusions

The results obtained in this study indicate that BoNT-A represents an effective and safe therapeutic option for pain relief in cases of M-TMDs, in addition to providing beneficial effects on mandibular function and patients' quality of life.

We observed a significant and sustained improvement in pain symptoms at 6 and 12 weeks after BoNT-A administration, along with a positive impact on functional limitations and psychosocial well-being, as assessed using validated clinical tools. Furthermore, the absence of relevant adverse effects reinforces the safety profile of the protocol adopted, which was based on a moderate dose and careful anatomical distribution.

These findings contribute to consolidating the existing scientific evidence on the efficacy of BoNT-A in the context of M-TMDs and suggest its possible role as part of a multidisciplinary therapeutic approach.

However, for robust validation of these results and the establishment of clear clinical guidelines, further studies with larger sample sizes, the inclusion of a placebo control group, longer follow-up, and comparisons with other therapeutic strategies are required. Future research should also consider evaluating the economic impact of this intervention and developing standardized clinical application protocols.

## Data Availability

The original contributions presented in the study are included in the article/Supplementary Material, further inquiries can be directed to the corresponding author.

## References

[1] Schiffman E Ohrbach R Truelove E Look J Anderson G Goulet JP Diagnostic criteria for temporomandibular disorders (DC/TMD) for clinical and research applications: recommendations of the International RDC/TMD Consortium Network* and Orofacial Pain Special Interest Group†. J Oral Facial Pain Headache. (2014) 28:6–27. 10.11607/jop.1151 24482784 PMC4478082

[2] Ohrbach R Greene C. Temporomandibular disorders: priorities for research and care. J Dent Res. (2022) 101:742–3. 10.1177/00220345211062047 35001698

[3] Wang X Yang Y Lin L Yao Q Zhang J. Obesity and temporomandibular joint disorders: a systematic review and meta-analysis. BMC Oral Health. (2023) 23:607. 10.1186/s12903-023-03322-2 37644424 PMC10466750

[4] Valesan LF Da-Cas CD Réus JC Denardin ACS Garanhani RR Bonotto D Prevalence of temporomandibular joint disorders: a systematic review and meta-analysis. Clin Oral Investig. (2021) 25:441–53. 10.1007/s00784-020-03710-w 33409693

[5] Minervini G Franco R Marrapodi MM Fiorillo L Cervino G Cicciù M. Prevalence of temporomandibular disorders in children and adolescents evaluated with diagnostic criteria for temporomandibular disorders: a systematic review with meta-analysis. J Oral Rehabil. (2023) 50:522–30. 10.1111/joor.13446 36912441

[6] Al-Hanbali LMS Burhan AS Hajeer MY Sultan K Nawaya FR. The effectiveness of interventions in reducing pain related to orthodontic separation: a systematic review and meta-analysis. Eur J Orthod. (2024) 46:cjad078. 10.1093/ejo/cjad078 38168817

[7] Kapos FP Exposto FG Oyarzo JF Durham J. Temporomandibular disorders: a review of current concepts in aetiology, diagnosis and management. Oral Surg. (2020) 13:321–34. 10.1111/ors.12473 34853604 PMC8631581

[8] Sójka A Stelcer B Roy M Mojs E Pryliński M. Is there a relationship between psychological factors and TMD? Brain Behav. (2019) 9:e01386. 10.1002/brb3.1360 31339236 PMC7649956

[9] Christidis N Al-Moraissi EA Al-Ak'hali MS Minarji N Zerfu B Grigoriadis A Psychological treatments for temporomandibular disorder pain-A systematic review. J Oral Rehabil. (2024) 51:1320–36. 10.1111/joor.13693 38616535

[10] Gupta AK Singh RK Narula V. Centric stabilization occlusal splints vs. other conservative therapies in the management of temporomandibular disorders: a systematic review and meta-analysis. Saudi Dent J. (2025) 37:52. 10.1007/s44445-025-00052-9 41091278 PMC12528636

[11] Armijo-Olivo S Pitance L Singh V Neto F Thie N Michelotti A. Effectiveness of manual therapy and therapeutic exercise for temporomandibular disorders: systematic review and meta-analysis. Phys Ther. (2016) 96:9–25. 10.2522/ptj.20140548 26294683 PMC4706597

[12] Ouanounou A Goldberg M Haas DA. Pharmacotherapy in temporomandibular disorders: a review. J Can Dent Assoc. (2017) 83:h7. 29513209

[13] Shehri ZG Alkhouri I Hajeer MY Haddad I Abu Hawa MH. Evaluation of the efficacy of low-dose Botulinum toxin injection into the masseter muscle for the treatment of nocturnal bruxism: a randomized controlled clinical trial. Cureus. (2022) 14:e32180. 10.7759/cureus.32180 36474649 PMC9719743

[14] Wie C Dunn T Sperry J Strand N Dawodu A Freeman J Cognitive behavioral therapy and biofeedback. Curr Pain Headache Rep. (2025) 29:23. 10.1007/s11916-024-01348-x 39786604

[15] Liu L Chen Q Lyu T Zhao L Miao Q Liu Y Effect of acupuncture for temporomandibular disorders: a randomized clinical trial. QJM. (2024) 117:647–56. 10.1093/qjmed/hcae094 38710498 PMC11537310

[16] Dunning J Butts R Bliton P Vathrakokoilis K Smith G Lineberger C Dry needling and upper cervical spinal manipulation in patients with temporomandibular disorder: a multi-center randomized clinical trial. Cranio. (2024) 42:809–22. 10.1080/08869634.2022.2062137 35412448

[17] Chen YW Chiu YW Chen CY Chuang SK. Botulinum toxin therapy for temporomandibular joint disorders: a systematic review of randomized controlled trials. Int J Oral Maxillofac Surg. (2015) 44:1018–26. 10.1016/j.ijom.2015.04.003 25920597

[18] Saini RS Ali Abdullah Almoyad M Binduhayyim RIH Quadri SA Gurumurthy V Bavabeedu S The effectiveness of botulinum toxin for temporomandibular disorders: a systematic review and meta-analysis. PLoS One. (2024) 19:e0300157. 10.1371/journal.pone.0300157 38483856 PMC10939295

[19] Le V Shah A Elgazzar R. Treatment of myofascial pain and dysfunction using botulinum toxin A: a prospective study. Tanta Dent J. (2024) 21:319–29. 10.4103/tdj.tdj_51_23

[20] Yacoub S Ons G Khemiss M. Efficacy of botulinum toxin type A in bruxism management: a systematic review. Dent Med Probl. (2025) 62:145–60. 10.17219/dmp/186553 40035138

[21] Sendra LA Azeredo Alves Antunes L Barboza EP. Use of botulinum neurotoxin type A in the management of primary bruxism in adults: an updated systematic review. J Prosthet Dent. (2024) 132:93–9. 10.1016/j.prosdent.2022.05.009 35779974

[22] Sidebottom AJ Patel AA Amin J. Botulinum injection for the management of myofascial pain in the masticatory muscles. A prospective outcome study. Br J Oral Maxillofac Surg. (2013) 51:199–205. 10.1016/j.bjoms.2012.07.002 22871559

[23] Foster L Clapp L Erickson M Jabbari B. Botulinum toxin A and chronic low back pain. A randomized, double-blind study. Neurology. (2001) 56:1290–3. 10.1212/wnl.56.10.1290 11376175

[24] Park J Park HJ. Botulinum toxin for the treatment of neuropathic pain. Toxins (Basel). (2017) 9(9):260. 10.3390/toxins9090260 28837075 PMC5618193

[25] Balanta-Melo J Toro-Ibacache V Kupczik K Buvinic S. Mandibular bone loss after masticatory muscles intervention with Botulinum toxin: an approach from basic research to clinical findings. Toxins (Basel). (2019) 11:84. 10.3390/toxins11020084 30717172 PMC6409568

[26] Raphael KG Janal MN Tadinada A Santiago V Sirois DA Lurie AG. Effect of multiple injections of botulinum toxin into painful masticatory muscles on bone density in the temporomandibular complex. J Oral Rehabil. (2020) 47:1319–29. 10.1111/joor.13087 32885475 PMC7693250

[27] Montaser MMS Elsokkary NH Shararah AEAI. Effect of botulinum toxin type A on masticatory function and musculoskeletal structure in rabbits. Sci Rep. (2025) 15:15323. 10.1038/s41598-025-97919-y 40312522 PMC12045985

[28] Wojtovicz EL Alvarez OM Lopez-Davis A Armijo-Olivo S. Botulinum toxin type A injection into the masticatory muscles and its effects on mandibular bone resorption and density: a systematic review. Clin Oral Investig. (2024) 28:1–17. 10.1007/s00784-024-05838-5 39123075

[29] Moher D Hopewell S Schulz KF Montori V Gøtzsche PC Devereaux PJ CONSORT 2010 Explanation and elaboration: updated guidelines for reporting parallel group randomised trials. Int J Surg. (2012) 10:28–55. 10.1136/bmj.c869 22036893

[30] Jaber ST Hajeer MY Burhan AS Latifeh Y. The effect of treatment with clear aligners versus fixed appliances on oral health-related quality of life in patients with severe crowding: a one-year follow-up randomized controlled clinical trial. Cureus. (2022) 14:e25472. 10.7759/cureus.25472 35663697 PMC9156343

[31] Delgado DA Lambert BS Boutris N McCulloch PC Robbins AB Moreno MR Validation of digital visual analog scale pain scoring with a traditional paper-based visual analog scale in adults. J Am Acad Orthop Surg Glob Res Rev. (2018) 2:e088. 10.5435/JAAOSGlobal-D-17-00088 30211382 PMC6132313

[32] Campos LA Peltomäki T Marôco J Campos JADB. Use of oral health impact profile-14 (OHIP-14) in different contexts: what is being measured? Int J Environ Res Public Health. (2021) 18:13412. 10.3390/ijerph182413412 34949018 PMC8703465

[33] Alhafi ZM Hajeer MY Latifeh Y Almusawi AOA Burhan AS Azizia T The impact of non-extraction orthodontic treatment on the oral-health-related quality of life between a modified aligner appliance with Ni-ti springs and the traditional fixed appliances: a randomized controlled clinical trial. Medicina (B Aires). (2024) 60:1139. 10.3390/medicina60071139 PMC11279390 39064568

[34] Ohrbach R Granger C List T Dworkin S. Preliminary development and validation of the jaw functional limitation scale. Community Dent Oral Epidemiol. (2008) 36:228–36. 10.1111/j.1600-0528.2007.00397.x 18474055

[35] Wilkie DJ Molokie RE Suarez ML Ezenwa MO Wang ZJ. Composite pain Index: reliability, validity, and sensitivity of a patient-reported outcome for research. Pain Med. (2015) 16:1341–8. 10.1111/pme.12703 25712169 PMC4504760

[36] Jadhao VA Lokhande N Habbu SG Sewane S Dongare S Goyal N. Efficacy of botulinum toxin in treating myofascial pain and occlusal force characteristics of masticatory muscles in bruxism. Indian J Dent Res. (2017) 28:493–7. 10.4103/ijdr.IJDR_125_17 29072209

[37] Guarda-Nardini L Manfredini D Salamone M Salmaso L Tonello S Ferronato G. Efficacy of botulinum toxin in treating myofascial pain in bruxers: a controlled placebo pilot study. Cranio. (2008) 26:126–35. 10.1179/crn.2008.017 18468272

[38] De la Torre Canales G Poluha RL Bonjardim LR Ernberg M Conti PCR. Botulinum toxin-A effects on pain, somatosensory and psychosocial features of patients with refractory masticatory myofascial pain: a randomized double-blind clinical trial. Sci Rep. (2024) 14:4201. 10.1038/s41598-024-54906-z 38378855 PMC10879180

[39] Montes-Carmona JF Gonzalez-Perez LM Infante-Cossio P. Treatment of localized and referred masticatory myofascial pain with Botulinum toxin injection. Toxins (Basel). (2020) 13:6. 10.3390/toxins13010006 33374687 PMC7822413

[40] Gonzalez-Perez LM Vera-Martin R Montes-Latorre E Torres-Carranza E Infante-Cossio P. Botulinum toxin and percutaneous needle electrolysis for the treatment of chronic masticatory myalgia. Toxins (Basel). (2023) 15:278. 10.3390/toxins15040278 37104216 PMC10144780

[41] Alam MK Abutayyem H Alzabni KMD Almuhyi NHS Alsabilah KAS Alkubaydan FST The impact of temporomandibular disorders on orthodontic management: a systematic review and meta-analysis. Cureus. (2023) 15:e44243. 10.7759/cureus.44243 37645665 PMC10461594

[42] Yap AU Lei J Liu C Fu KY. Characteristics of painful temporomandibular disorders and their influence on jaw functional limitation and oral health-related quality of life. J Oral Rehabil. (2024) 51:1748–58. 10.1111/joor.13768 38845181

[43] De la Torre Canales G Poluha RL Pinzón NA Da Silva BR Almeida AM Ernberg M Efficacy of Botulinum toxin type-A I in the improvement of mandibular motion and muscle sensibility in myofascial pain TMD subjects: a randomized controlled trial. Toxins (Basel). (2022) 14:441. 10.3390/toxins14070441 35878179 PMC9323061

[44] De Carli BM Magro AK Souza-Silva BN Matos Fde S De Carli JP Paranhos LR The effect of laser and botulinum toxin in the treatment of myofascial pain and mouth opening: a randomized clinical trial. J Photochem Photobiol B. (2016) 159:120–3. 10.1016/j.jphotobiol.2016.03.038 27045280

[45] Kim SR Chang M Kim AH Kim ST. Effect of Botulinum toxin on masticatory muscle pain in patients with temporomandibular disorders: a randomized, double-blind, placebo-controlled pilot study. Toxins (Basel). (2023) 15:597. 10.3390/toxins15100597 37888628 PMC10610636

[46] Nixdorf DR Heo G Major PW. Randomized controlled trial of botulinum toxin A for chronic myogenous orofacial pain. Pain. (2002) 99:465–73. 10.1016/S0304-3959(02)00240-3 12406522

[47] Li K Tan K Yacovelli A Bi WG. Effect of botulinum toxin type A on muscular temporomandibular disorder: a systematic review and meta-analysis of randomized controlled trials. J Oral Rehabil. (2024) 51:886–97. 10.1111/joor.13648 38151884

[48] De La Torre Canales G Câmara-Souza MB Muñoz Lora VRM Guarda-Nardini L Conti PCR Rodrigues Garcia RM Prevalence of psychosocial impairment in temporomandibular disorder patients: a systematic review. J Oral Rehabil. (2018) 45:881–9. 10.1111/joor.12685 29972707

[49] Bitiniene D Zamaliauskiene R Kubilius R Leketas M Gailius T Smirnovaite K. Quality of life in patients with temporomandibular disorders. A systematic review. Stomatologija. (2018) 20:3–9. 29806652

[50] Pigozzi LB Pereira DD Pattussi MP Moret-Tatay C Irigaray TQ Weber JBB Quality of life in young and middle age adult temporomandibular disorders patients and asymptomatic subjects: a systematic review and meta-analysis. Health Qual Life Outcomes. (2021) 19:83. 10.1186/s12955-021-01727-7 33691709 PMC7945303

[51] Zhang Q Wu W Fan Y Li Y Liu J Xu Y The safety and efficacy of botulinum toxin A on the treatment of depression. Brain Behav. (2021) 11:e2333. 10.1002/brb3.2333 34423572 PMC8442586

[52] Wollmer MA Magid M Kruger THC Finzi E. Treatment of depression with Botulinum toxin. Toxins (Basel). (2022) 14:383. 10.3390/toxins14060383 35737044 PMC9231293

[53] Blumenfeld AM Tepper SJ Robbins LD Manack Adams A Buse DC Orejudos A Effects of onabotulinumtoxinA treatment for chronic migraine on common comorbidities including depression and anxiety. J Neurol Neurosurg Psychiatry. (2019) 90:353–60. 10.1136/jnnp-2018-319290 30630956 PMC6518474

[54] Magid M Reichenberg JS Poth PE Robertson HT LaViolette AK Kruger TH Treatment of major depressive disorder using botulinum toxin A: a 24-week randomized, double-blind, placebo-controlled study. J Clin Psychiatry. (2014) 75:837–44. 10.4088/JCP.13m08845 24910934

[55] Magid M Keeling BH Reichenberg JS. Neurotoxins: expanding uses of neuromodulators in medicine–Major depressive disorder. Plast Reconstr Surg. (2015) 136:111S–9. 10.1097/PRS.0000000000001733 26441090

[56] Sirri MR Hajeer MY Baba F Aljabban O Burhan AS Namera MO. Root resorption associated with minimally invasive surgical acceleration of orthodontic tooth movement: a systematic review. Eur J Orthod. (2025) 47:cjaf035. 10.1093/ejo/cjaf035 40556468

[57] Delcanho R Val M Guarda Nardini L Manfredini D. Botulinum toxin for treating temporomandibular disorders: what is the evidence? J Oral Facial Pain Headache. (2022) 36:6–20. 10.11607/ofph.3023 35298571 PMC10586579

[58] Kharraz RH Mushan NA Alshehri GM Dhaen MM AlGalal HA Khashfa RA A comparative analysis of Botulinum toxin use versus other therapies for temporomandibular disorders: a systematic review. Cureus. (2024) 16:e70389. 10.7759/cureus.70389 39469401 PMC11515690

[59] De la Torre Canales G Alvarez-Pinzon N Muñoz-Lora VR Vieira Peroni L Farias Gomes A Sánchez-Ayala A Efficacy and safety of Botulinum toxin type A on persistent myofascial pain: a randomized clinical trial. Toxins (Basel). (2020) 12:395. 10.3390/toxins12060395 32549196 PMC7354430

[60] Matak I Bölcskei K Bach-Rojecky L Helyes Z. Mechanisms of Botulinum toxin type A action on pain. Toxins (Basel). (2019) 11:459. 10.3390/toxins11080459 31387301 PMC6723487

[61] Rodríguez-Gimillo P Valverde-Navarro A Margaix-Muñoz M Poveda-Roda R Delgado-Navarro C Puig-Bernabeu J. Lateral pterygoid muscle ultrasound-guided injection: a technical note. J Stomatol Oral Maxillofac Surg. (2024) 125:101547. 10.1016/j.jormas.2023.101547 37394100

[62] De la Torre Canales G Câmara-Souza MB Ernberg M Al-Moraissi EA Grigoriadis A Poluha RL Botulinum toxin-A for the treatment of myogenous temporomandibular disorders: an Umbrella review of systematic reviews. Drugs. (2024) 84:779–809. 10.1007/s40265-024-02048-x 38900335 PMC11289222

[63] Park HU Kim BI Kang SM Kim ST Choi JH Ahn HJ. Changes in masticatory function after injection of botulinum toxin type A to masticatory muscles. J Oral Rehabil. (2013) 40:916–22. 10.1111/joor.12111 24237358

[64] Nestor MS Arnold D Fischer DL. The mechanisms of action and use of botulinum neurotoxin type A in aesthetics: key clinical postulates II. J Cosmet Dermatol. (2020) 19:2785–804. 10.1111/jocd.14108 32866999 PMC7693297

[65] de Souza Nobre B Rezende L Barbosa Câmara-Souza M. Exploring botulinum toxin’s impact on masseter hypertrophy: a randomized, triple-blinded clinical trial. Sci Rep. (2024) 14:14522. 10.1038/s41598-024-65395-5 38914688 PMC11196657

[66] Moussa MS Bachour D Komarova SV. Adverse effect of botulinum toxin-A injections on mandibular bone: a systematic review and meta-analysis. J Oral Rehabil. (2024) 51:404–15. 10.1111/joor.13590 37668276

